# Caries prevalence in children from Valencia (Spain) using ICDAS II criteria, 2010

**DOI:** 10.4317/medoral.19890

**Published:** 2014-09-30

**Authors:** José M. Almerich-Silla, Teresa Boronat-Ferrer, José M. Montiel-Company, José E. Iranzo-Cortés

**Affiliations:** 1 Tenured Lecturer. Facultad de Medicina y Odontología. Departamento de Estomatología. Universitat de València; 2 Associate Lecturer. Facultad de Medicina y Odontología. Departamento de Estomatología. Universitat de València; 3 Post-Doctoral Assistant Lecturer. Facultad de Medicina y Odontología. Departamento de Estomatología. Universitat de València; 4 PhD Student. Facultad de Medicina y Odontología. Departamento de Estomatología. Universitat de València

## Abstract

Objectives: To assess the oral health status of the child population and its evolution over the 2004-2010 period. Study Design: A descriptive cross-sectional study of the entire schoolchild population of the Valencia region of Spain was conducted using cluster sampling. Seventy schools were selected. The sample size was 1373 pupils, aged 6, 12 and 15 years. The children were examined in November and December 2010, at their schools, by 6 examiners calibrated in the ICDAS II method. The descriptive statistics, comparison of means and comparison of proportions (*p*<0.05) were recorded. Results: The caries prevalence (ICDAS 4-6>0) was 30% in primary dentition at 6 years and 37.7% and 43.6% in permanent dentition at 12 and 15 years respectively. At 6, 12 and 15 years, the df.t/DMF. TICDASII 4-6 scores were 0.98, 0.83 and 1.08, the df.s/DMF.SICDASII 4-6 scores were 1.43, 1.27 and 1.64 and the care index results were 14.3%, 59% and 71.3% respectively. Conclusions: Both the caries indices (df.t at 6 years and DMF.T at 12 and 15) and caries prevalence have improved, as the values obtained were lower than in 2004. Using the comparison at 95% CI, between both years, the improvement was only noticeable in the 15 year-old group. The care index continued to be low at 6 years of age but higher values than in 2004 were found at 12 and 15 years. Social class continued to influence the child caries indicators.

** Key words:**Caries prevalence, schoolchildren, ICDAS II, cross-sectional survey.

## Introduction

Basic oral health surveys provide a sound basis for assessing the current and future oral health care needs of a population, as they provide reliable data on which to base oral health programmes and planning for suitable numbers of the appropriate types of dental care personnel.

In the Valencia region of Spain, epidemiological surveys of the child population were conducted in 1986, 1991, 1998 and 2004 (1), in the latter two cases following the methods proposed by the WHO in 1997.

The trend towards a progressive reduction in caries prevalence observed in the surveys conducted over the past 15 years prompted the authors to propose the use of more sensitive indices that identify the different stages of dental caries lesions that precede cavitation. Consequently, it was suggested that the ICDAS II (International Caries Detection and Assessment System) criteria should be used for diagnosing and coding caries lesions.

ICDAS II is a caries diagnosis and assessment system that was devised with the aim of reaching consensus on the clinical criteria for detecting and coding the initial stages of caries, in which changes of colour can be observed before cavitation occurs. The new epidemiological trends concerning dental caries make it necessary to introduce ICDAS II as a caries diagnosis criterion in order to codify incipient caries lesions in both epidemiological and clinical situations. Its use is still limited but can already be found in several recent studies (2-6).

The oral health study of children in the Valencia region of Spain in the year 2010, presented here below, is the first epidemiological study conducted in a Spanish region with this diagnostic criterion. Its objective was to ascertain the current child oral health situation and assess its evolution over the 2004-2010 period, using ICDAS II criteria.

## Material and Methods

-Sample size

The study population of this cross-sectional descriptive study was the entirety of the schoolchild population of the Valencia region of Spain. Cluster sampling was provided by the Public Health authority of Comunidad Valenciana. From the 1200 schools in the region, 70 were selected at random. The study sample size was 1373 children in the 6, 12 and 15 year-old age cohorts.

-Prior calibration

The examiners were calibrated before the study began. The 6 examiners were handed an examination manual that set out the diagnostic criteria and the data that should be recorded and showed the examination forms that would be used in the study. Once they had studied its content, several theoretical sessions were held to exchange criteria and answer questions.

Firstly, the examiners followed the on-line ICDAS II calibration course. They then performed a calibration exercise in which they each examined 10 children and compared their results with those of an experienced examiner who had taken part in several similar surveys in previous years, who acted as the gold standard. The 6 examiners’ weighted inter-examiner Kappa scores in relation to the gold standard were 0.76, 0.78, 0.83, 0.85, 0.85 and 0.91. The 3 examiners with the highest scores acted as examiners and the other 3 as recorders, forming 3 examination teams.

-Informed consent 

The children’s parents or tutors received detailed information on the ex-aminations to be performed and gave their consent by signing the authorisation. The examinations were carried out after receiving the authorisation signed by the parents/tutors. Sampling loss by this cause was less than 10%. The study was approved by the University of Valencia’s Ethics Committee.

-Materials

The examination instruments employed were a WHO-type periodontal probe, a no. 5 plain mouth mirror and a portable air compressor to dry the teeth. New disposable latex gloves and face masks were used for each examination.

-Examinations

All the examinations were carried out at the selected schools, in the place decided by the head teacher of each school. The study was carried out in November and December 2010 .

The examinations took place with the child sitting on a chair, with his or her head extended, and the examiner sitting opposite. While the examiner proceeded with the examination, the recorder completed the assessment form.

The examination form used included identification or personal data and data on the oral examination.

-Caries diagnosis criteria 

The ICDAS II system was used (https://www.icdas.org/). All the dental surfaces were initially examined when wet. The surfaces were then dried with air for five seconds to observe any changes in colour.

When using the ICDAS II criteria for epidemiological studies, a cutoff point in the ICDAS II coding scale equivalent to the WHO caries definition has to be decided in order to ensure comparability between the data from the new studies and those obtained in previous studies using the WHO criteria. In the present study, for purposes of comparison with the WHO criteria, the cutoff point for grouping the ICDAS II codes was set at code 4, as established by the ICDAS II criteria themselves, in which grades 4, 5 and 6 (ICDAS II 4-6) are considered equivalent to the WHO definition of caries.

-Study variables

The variables calculated for the study were caries prevalence (ICDAS 1-6>0 and ICDAS 4-6>0), df.tICDAS II 1-6, df.tICDAS II 4-6, df.sICDAS II 1-6, df.sICDAS II 4-6, DMF.TICDAS II 1-6, DMF.TICDAS II 4-6, DMF.SICDAS II 1-6 and DMF.SICDAS II 4-6. The care, morbidity, mortality and significant caries (SiC) indices were calculated from the codes 4-6 results.

The assessment of social class was made according to the classification proposed by Domingo *et al*. (7), based on the parents’ occupation, in which the social class of the child is considered to be the higher of those of the two parents.

For the present study, the categories were grouped into high, middle and low social class. Classes I and II were considered high social class, III middle class and IV and V low social class.

-Statistical analysis

Each examining team entered the data from each form into a Microsoft® Access® database, in the same format as on the paper form for ease of processing.

The statistical analysis was conducted with SPSS v.18.0® software. Descriptive statistics with means, proportions and their 95% confidence intervals were calculated. In the bivariate statistics, Student’s t-test and ANOVA were used to compare means and the Chi squared test to compare proportions. The significance level was <0,05.

## Results

The size of the valid study sample was 1373 children. For the sample size and the caries prevalence encountered, at a 95% confidence level, the precision (ε) of the study was 0.041 for the 6 year-old group, 0.045 for the 12 year-olds and 0.047 for the 15 year-olds.

The caries prevalence (df.t /DMF.TICDAS II 4-6>0) was 30% at 6 years of age, 37.7% at 12 years and 43.6% at 15 years. The caries prevalence including codes 1 to 3 (df.t/DMF.TICDAS II 1-6>0) is shown in  Table 1.

**Table 1:**
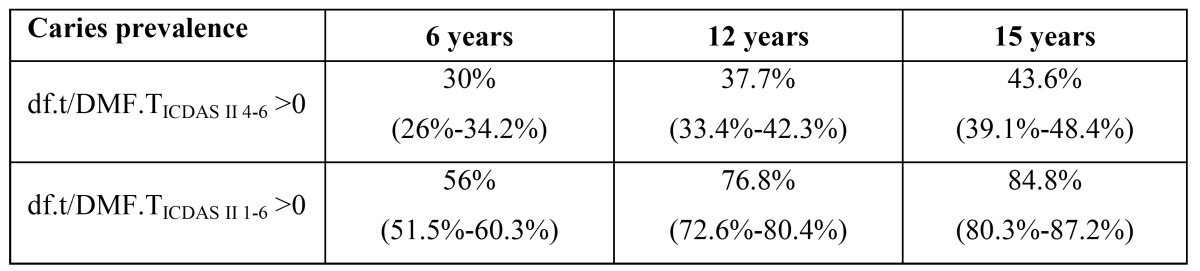
caries prevalence by age (CI 95%).

The df.tICDAS II 1-6 index at 6 years was 2.13. The DMF.TICDAS II 1-6 index was 3.46 at 12 years and 4.74 at 15 years of age ( Table 2). The number of initial caries lesions (codes 1-3) was 1.15 at 6 years, 2.63 at 12 years and 3.65 at 15 years.

**Table 2:**
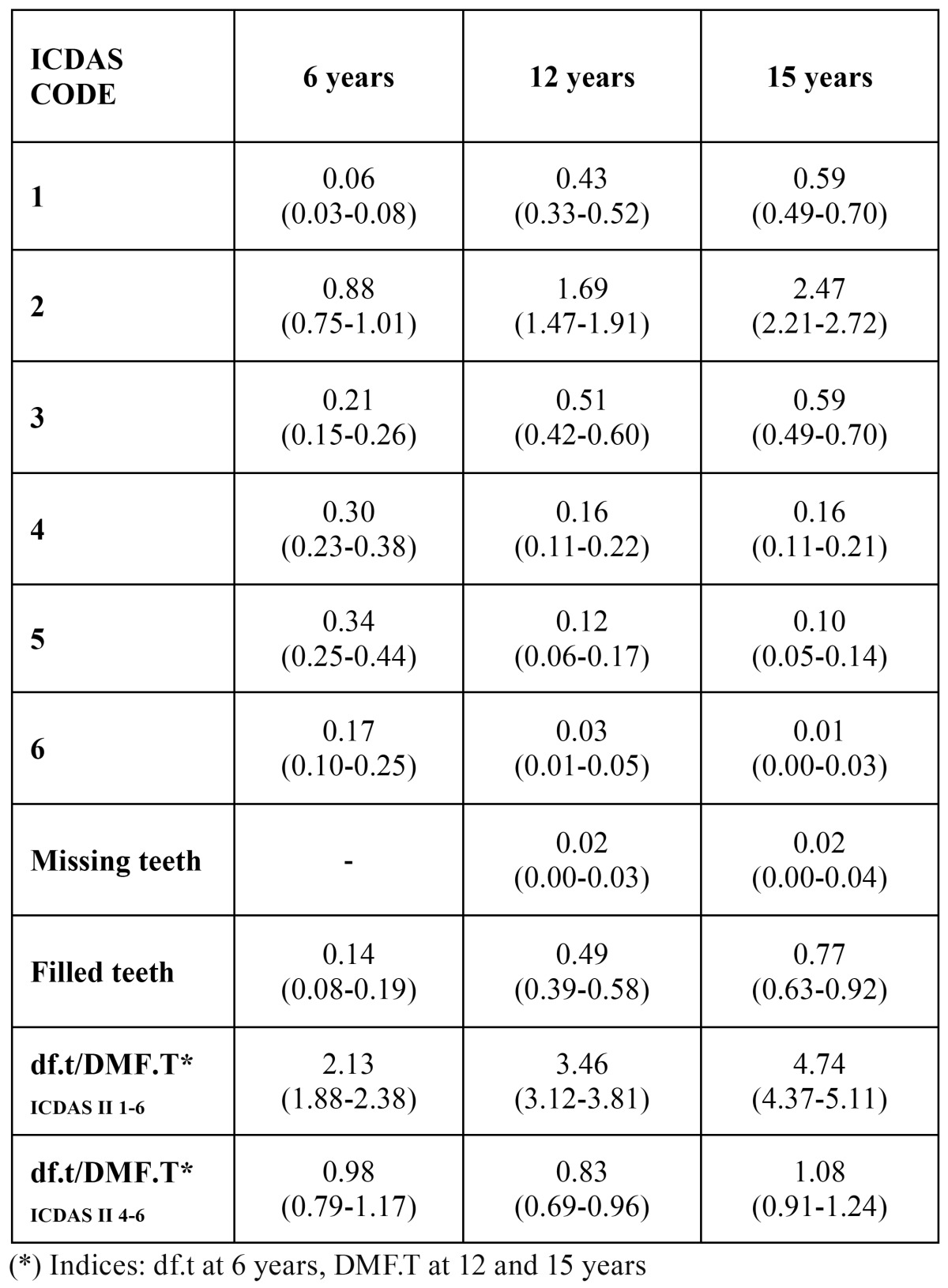
df.t/DMF.T indices and codes by age (CI 95%).

The df.sICDAS II 1-6 index at 6 years was 1.77. The DMF.SICDAS II 1-6 index at 12 and 15 years was 4.45 and 5.87 respectively. The number of initial caries lesions (codes 1-3) was 1.33 at 6 years, 3.18 at 12 years and 4.23 at 15 years ( Table 3).

**Table 3:**
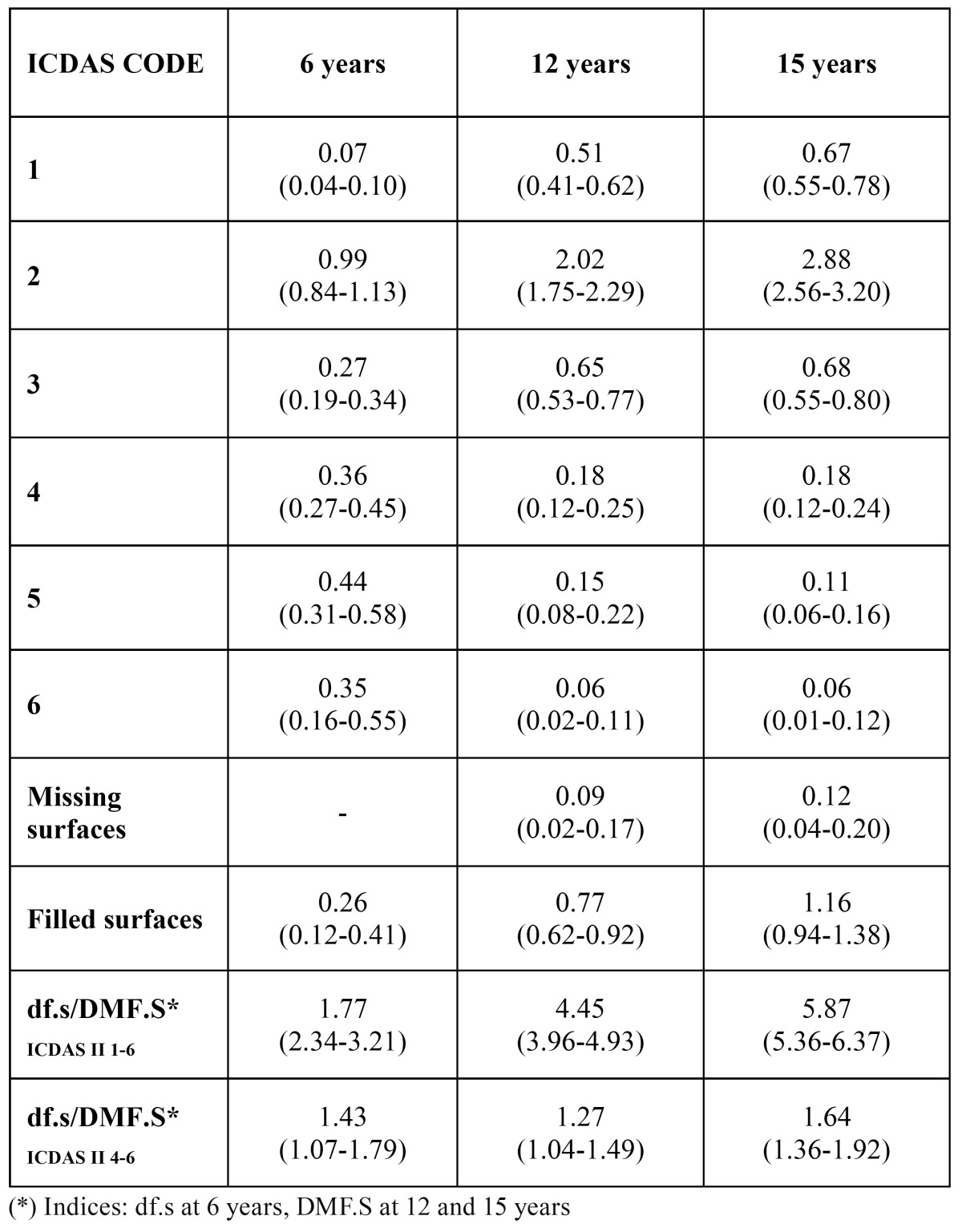
df.s/DMF.S indices and codes by age (CI 95%).

On calculating these indices from the cut-off point proposed by ICDAS as equivalent to the WHO criteria, at 6 years of age the df.tICDAS 4-6 index was 0.98 and the df.sICDAS 4-6 index was 1.43. At 12 years the DMF.TICDAS 4-6 was 0.83 and the DMF.SICDAS 4-6 was 1.27. Lastly, at 15 years of age the DMF.TICDAS 4-6 was 1.08 and the DMF.SICDAS 4-6 was 1.64.

The same cutoff point was used to calculate the care index, which in primary dentition at the age of 6 years was 14.3%. The care index for permanent teeth was 59.0% for the 12 year-olds and 71.3% for the 15 year-olds. The morbidity index (taking caries as comprising ICDAS II codes 4, 5 and 6) in primary dentition at 6 years of age was 84.7%. The same index for permanent dentition at 12 and 15 years was 38.5% and 25.9% respectively. The dental mortality index was 2.4% at 12 years and 1.8% at 15 years of age. The SiCICDAS 4-6 score for the 12-year old group was 2.36 (95% CI 2.10-2.61).

The distribution of the sample by social class was 42.4% low social class, 37.7% middle-class and 14.2% high social class. The class of 5.6% of the sample was coded as ‘not recorded’.

An inversely proportional association was found between caries and social class in all the age groups, although it was not significant ( Table 4). The same tendency was found in the caries indices, where the diffe-rence was statistically significant in primary dentition at 6 years of age ( Table 5).

**Table 4:**
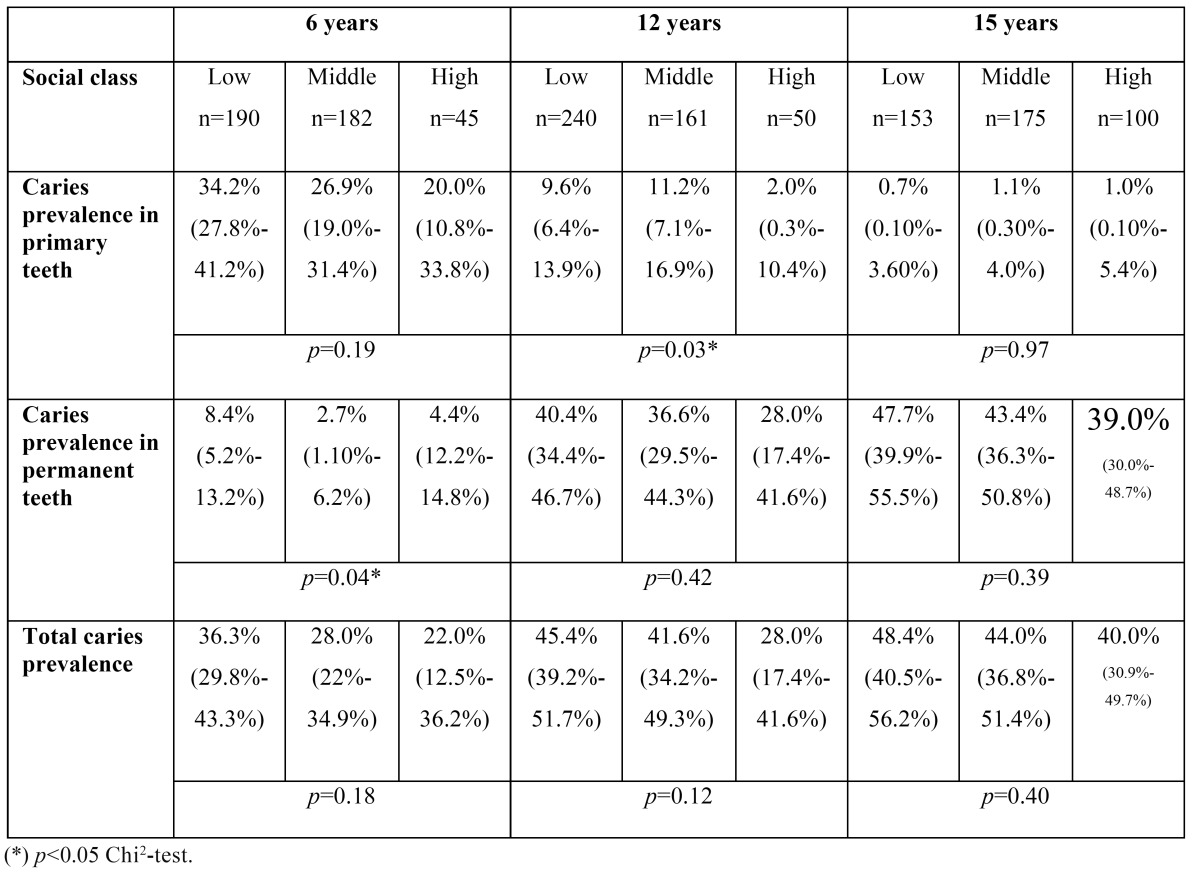
df.t/DMF.T indices and codes by age (CI 95%).

**Table 5:**
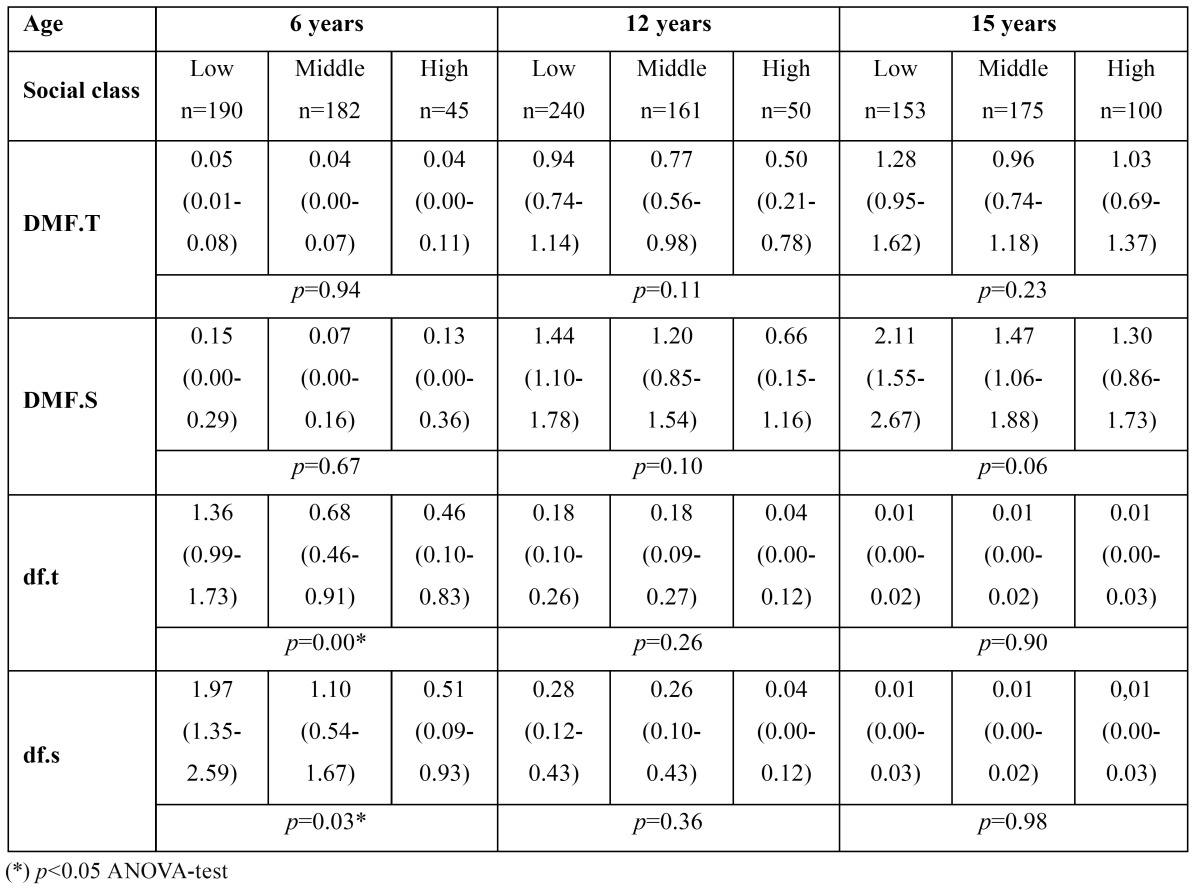
Caries indices by social class and age (CI 95%).

## Discussion

Cluster sampling used was provided by the public health authorities of Valencia. This could be a bias compared with simple random sampling but the nature of fieldwork makes it advisable to use, according to the methodology used in most studies on children.

One distinctive feature of the present study is that it recorded incipient caries with the ICDAS II system. Indeed, this is the first epidemiological survey conducted in a Spanish region to use ICDAS II diagnostic criteria.

ICDAS II was devised as a tool for diagnosing caries in both epidemiological and clinical situations. The new trends in dental caries epidemiology make it necessary to introduce ICDAS II as a diagnostic criterion in epidemiological studies and in daily clinical practice. Some studies have examined certain drawbacks in the application of this index, particularly the time factor and the examiner’s skills. The examination time is greater when ICDAS II is used because all the teeth and all their surfaces have to be coded both when wet and again after drying. A study that quantified the time employed concluded that recording with the ICDAS II criteria required double the time compared to the WHO criteria. The examiners also have to be trained in the use and correct application of the more complex ICDAS II codes, particularly the coding of the initial stages of caries. Nevertheless, it is logical to consider that aspects such as the time needed for the examination and the skill of the examiner, which are now considered limiting factors, will improve with practice and the more frequent employment of this system as dentists acquire knowledge and training and it comes into widespread use.

Another drawback when beginning to use ICDAS II for epidemiological studies is that few studies have been carried out with it, and none in Spain, so to compare the data from the new studies with those obtained in previous studies using the WHO criteria it is necessary to set a cutoff point in the ICDAS II coding scale that is equivalent to the WHO caries definition. There is some controversy concerning equivalents with the WHO criterion, as some authors (5,6,8) consider that this cutoff point may be found at code 3, whereas the ICDAS II criterion itself states that codes 4, 5 and 6 are equivalent to the WHO definition of caries. The present study sets the ICDAS II cutoff point for comparison with the WHO criteria at code 4.

Despite these drawbacks, the use of new caries criteria, specifically the ICDAS II system, is amply justified in order to adapt to the new epidemiological standards concerning dental caries.

Because the df.tICDAS II 1-6, df.sICDAS II 1-6, DMF.TICDAS II 1-6 and DMF.SICDAS II 1-6 results include incipient and precavitation carious lesions, these indices were high in comparison with those for the corresponding WHO criteria. At 12 years of age the mean incipient lesions per capita were 2.63, compared to a mean of 0.31 cavitated lesions according to the WHO criteria. In other words, the 12 year-olds had 8 incipient lesions for each cavitated lesion. The 6 year-old group’s mean non-cavitated and cavitated lesions were 1.45 and 0.51 respectively, that is to say, about 3 non-cavitated lesions for every cavitated one. This has also been observed in a study conducted in Brazil, where the df.t index was 1.87 according to the WHO and 15.46 according to the ICDAS II criteria (6), so 8 incipient lesions were found for each cavitated lesion. In another study in Colombia (5), the greatest caries prevalence was again found in non-cavitated lesions. The results of examinations for dental caries also increase when other diagnostic tests are performed, as in a study carried out in Iceland (4), in which visual examination at the ages of 12 and 15 years was complemented by intra-oral radiography. The number of non-cavitated caries lesions increased considerably on including the radiographic record.

Consequently, despite the tendency for caries prevalence to fall in recent decades according to the WHO diagnostic criteria, caries levels remain high when initial carious lesions are counted, on the clinical principle that these lesions also constitute caries. In view of the above studies, a new dental caries treatment protocol that places greater importance on preventing caries than on restoration should be drawn up.

On comparing the data from the present study with those of previous surveys in the Valencia region of Spain, the caries prevalence in primary dentition at 6 years of age was 30% in 2010. Similar results were obtained in the surveys of 1998 and 2004 (1), which produced almost identical values (32.8% and 32.2% respectively). In permanent dentition, the 2010 survey found caries prevalence rates of 37.7% at 12 years of age and 43.6% at 15 years. Both results were lower than in previous surveys in this region. In 2004 the rate was 42.5% at 12 years and 55.9% at 15 years, so the 2010 survey shows a clear drop in caries prevalence.

From the values obtained in the 2010 study, caries indices in line with the WHO criteria have also undergone changes. The df.tICDAS II 4-6 at 6 years was 0.98 in 2010 and 1.08 in 2004. At 12 years, the DMF.TICDAS 4-6 was 0.83 in 2010 and 1.07 in 2004. At 15 years, the same index was 1.08 in 2010 and 1.84 in 2004.

These data show that the caries index results have continued to fall since the study carried out in 2004 in the Valencia region. In comparison with the survey conducted in 1998, the indices remained at similar levels at the age of 6 years but tended to fall at 12 years. However, it was the 15 year-old group that exhibited this trend to a significantly greater degree between 1998 and 2010.

On comparing these results with studies conducted in other countries, results in Colombia (5) for children aged 2.5 to 4 years showed a caries prevalence rate of 74.9%, compared to 56% at 6 years of age in the 2010 survey of the Valencia region. The df.sICDAS II 1-6 value in the present study was 1.77 at 6 years, compared to 7.7 in Colombia and 0.53 in the Icelandic study (4). The df.tICDAS II 1-6 value at the same age was 2.13 in the present study, compared to 0.41 in Iceland.

In the present study the DMF.TICDAS II 1-6 value was 3.46 for the 12 year-old group and 4.74 for the 15 year-olds. The values in the Icelandic study were 2.82 and 4.43 respectively. DFT.SICDAS II 1-6 was 4.45 at 12 years and 5.87 at 15 years in the present study. In Iceland the values were 4.83 and 8.66 respectively.

The significant caries index (SiC) of the 12-year-olds was 2.36 in 2010. Compared with the 2004 value of 2.94, this indicates that dental caries in the Valencia region continues to be concentrated in a small percentage of the population. The Icelandic study obtained a value of 3.7 for this index.

In the present study, the care index in primary dentition at 6 years gave a value of 14.3%. The care index in permanent dentition was 59% at 12 years and 71.3% at 15 years. These figures are far higher than in 2004, when the care index was 32.7% at 12 years and 45% at 15 years.

## Conclusions

Both the caries indices (df.t ICDAS II 4-6 at 6 years and DMF.T ICDAS II 4-6 at 12 and 15) and caries prevalence have improved, as the values obtained in 2010 were lower than in 2004. Using the comparison at 95% CI, between both years, the improvement was only noticeable in the 15 year-old group.

The care index continued to be low at 6 years of age but higher values than in 2004 were found at 12 and 15 years. Social class continued to be an influential factor for the child caries indicators.

From these results it may be concluded that the child population of the Valencia region of Spain is meeting the 2015/2020 health objectives for these caries indicators proposed by the Spanish Public Oral Health Society (SESPO), (9).

Lastly, registering incipient carious lesions according to the ICDAS II criteria offers a new perspective of the disease from the initial and precavitation stages, pointing to the need for a new treatment protocol aimed at prevention.

## References

[1] Almerich Silla JM, Montiel Company JM (2006). Oral health survey of the child population in the Valencia Region of Spain (2004). Med Oral Patol Oral Cir Bucal.

[2] Bonner BC, Bourgeois DM, Douglas GV, Chan K, Pitts NB (2011). The feasibility of data collection in dental practices, using codes for the International Caries De-tection and Assessment System (ICDAS), to allow European general dental practitioners to monitor dental caries at local, national, and international levels. Prim Dent Care.

[3] Reisine S, Tellez M, Willem J, Sohn W, Ismail A (2008). Relationship between care-giver's and child's caries prevalence among disadvantaged African Americans. Community Dent Oral Epidemiol.

[4] Agustsdottir H, Gudmundsdottir H, Eggertsson H, Jonsson SH, Gudlaugsson JO, Saemundsson SR (2010). Caries prevalence of permanent teeth: a national survey of children in Iceland using ICDAS. Community Dent Oral Epidemiol.

[5] Cadavid AS, Lince CM, Jaramillo MC (2010). Dental caries in the primary dentition of a Colombian population according to the ICDAS criteria. Braz Oral Res.

[6] de Amorim RG, Figueiredo MJ, Leal SC, Mulder J, Frencken JE (2012). Caries ex-perience in a child population in a deprived area of Brazil, using ICDAS II. Clin Oral Investig.

[7] Domingo Salvany A, Marcos Alonso J (1989). Proposal of an indicator of "social class" based on the occupation. Gac Sanit.

[8] Iranzo-Cortes JE, Montiel-Company JM, Almerich-Silla JM (2013). Caries diagnosis: agreement between WHO and ICDAS II criteria in epidemiological surveys. Community Dent Health.

[9] Bravo M, Cortes J, Casals E, Llena C, Almerich-Silla JM, Cuenca E (2009). Basic oral health goals for Spain 2015/2020. Int Dent J.

